# New Cancer Immunotherapy Agents in Development: a report from an associated program of the 31^st^Annual Meeting of the Society for Immunotherapy of Cancer, 2016

**DOI:** 10.1186/s40425-017-0253-2

**Published:** 2017-06-20

**Authors:** Prasad S. Adusumilli, Edward Cha, Mark Cornfeld, Thomas Davis, Adi Diab, Thomas W. Dubensky, Elizabeth Evans, Jane L. Grogan, Bryan A. Irving, Rom S. Leidner, Shane A. Olwill, Patrick Soon-Shiong, Frederic Triebel, David Tuck, Adrian Bot, Roger D. Dansey, Charles G. Drake, Gordon J. Freeman, Ramy Ibrahim, Salil Patel, Daniel S. Chen

**Affiliations:** 1 Center for Cell Engineering; Thoracic Service, Department of Surgery, Memorial Sloan Kettering Cancer Center, 1275 York Avenue, New York, NY 10065 USA; 2 Genentech, 1 DNA Way, South San Francisco, CA 94080 USA; 3 Idera Pharmaceuticals, 505 Eagleview Blvd, Ste 212, Exton, PA 19341 USA; 4 Celldex Therapeutics, Inc, 119 Fourth Avenue, Needham, MA 02494 USA; 5 University of Texas MD Anderson Cancer Center, 1515 Holcomb Blvd, Unit 430, Houston, TX 77030 USA; 6 Aduro Biotech, Inc, 740 Heinz Avenue, Berkeley, CA 94710 USA; 7 Vaccinex, 1895 Mount Hope Avenue, Rochester, NY 14620 USA; 8 Genentech, 1 DNA Way, 13-2079, South San Francisco, CA 94080 USA; 9 Cytomx Therapeutics, 151 Oyster Point Blvd, South San Francisco, CA 94080 USA; 10 Earle A. Chiles Research Institute, Providence Cancer Center, 4805 NE Glisan St, Suite 2N35, Portland, OR 97213 USA; 11 Pieris Pharmaceuticals GmbH, Lise-Meitner-Str 30, Freising, 85354 Germany; 12 NantKwest, Inc, 9920 Jefferson Blvd, Culver City, CA 90232 USA; 13 Immutep S.A.S., Parc Club Orsay, 2 Rue Jean Rostand, Orsay, 91893 France; 14 Curis, 4 Maguire Road, Lexington, MA 02421 USA; 15 Kite Pharma, Inc, 2225 Colorado Ave, Santa Monica, CA 90404 USA; 16 Merck & Co., Inc, 126 E. Lincoln Avenue, RY34-B270, Rahway, NJ 07065 USA; 17 Division of Hematology/Oncology, Columbia University Medical Center, 177 Fort Washington Avenue, Suite 6GN-435, 10032 New York, NY USA; 18 Dana-Farber Cancer Institute, 450 Brookline Avenue, Boston, MA 02215 USA; 19 Parker Institute for Cancer Immunotherapy, 1 Letterman Drive, Suite D3500, San Francisco, CA 94129 USA; 20 Bristol-Myers Squibb, 3401 Princeton Pike, Lawrenceville, NJ 08648 USA; 21 Genentech/Roche, 1 DNA Way, South San Francisco, CA 94080 USA

**Keywords:** Immunotherapy, Cancer, Checkpoint inhibitors, PD-1, Combination therapy

## Abstract

This report is a summary of ‘New Cancer Immunotherapy Agents in Development’ program, which took place in association with the 31st Annual Meeting of the Society for Immunotherapy of Cancer (SITC), on November 9, 2016 in National Harbor, Maryland. Presenters gave brief overviews of emerging clinical and pre-clinical immune-based agents and combinations, before participating in an extended panel discussion with multidisciplinary leaders, including members of the FDA, leading academic institutions and industrial drug developers, to consider topics relevant to the future of cancer immunotherapy.

## Meeting Summary

In the wake of an unprecedented number of U.S. Food and Drug Administration (FDA) approvals for cancer immunotherapy agents, particularly immune checkpoint inhibitors, the field is poised for further advancement. An associated program of SITC’s 31^st^ Annual Meeting in November 2016, the ‘New Cancer Immunotherapy Agents in Development’ session was organized through collaboration between the SITC Annual Program Committee and the SITC Industry Committee. The goal of the program was to provide an opportunity to address challenges faced by experts in industry, government, and academia who are working to deliver better outcomes for patients with cancer. The recent focus on combination therapies has increased the complexity of this task and raises important mechanistic considerations as to how each additional agent affects the underlying biology of the cancer as well as the immune system of the individual. Moreover, logistical aspects of the application of cancer immunotherapy such as the use of biomarkers, optimal duration of treatment, determining the most appropriate clinical endpoints, and how many drugs to use in combination have yet to be determined.

To facilitate the conversation about drugs on the cancer immunotherapy horizon, program co-chairs Adrian Bot, MD, PhD (Kite Pharma, Inc.), Daniel S. Chen, MD, PhD (Genentech/Roche), Roger D. Dansey, MD (Merck & Co., Inc.), Ramy Ibrahim, MD (The Parker Institute for Cancer Immunotherapy), and Salil Patel, PhD (Bristol-Myers Squibb) divided the program into three sessions: (1) Clinical New Agents in Development; (2) Pre-Clinical New Agents in Development; and (3) Strategic Considerations of Combinations and Biomarkers in New Agent Development. The third session closed the program with an extended panel discussion featuring expert representatives from clinical, translational, and government backgrounds to provide unique perspectives on the development of cancer immunotherapeutics. This report summarizes key topics presented by the invited speakers and panel discussants.

## Clinical New Agents in Development

Elizabeth Evans, PhD (Vaccinex, Inc.) presented pre-clinical data supporting the combination of anti-semaphorin 4D (SEMA4D) with ipilimumab (anti-CTLA-4) or anti-PD-1/PD-L1 agents. SEMA4D is a guidance molecule capable of regulating the migration and differentiation of cells expressing its receptor. Expressed on tumor cells and immune cells at the invasive tumor margin, SEMA4D inhibits the migration of antigen presenting cells (APC) and prevents immune cells from infiltrating the tumor. Antibody blockade of SEMA4D facilitated the ability of functional tumor-specific CD8+ T cells and dendritic cells (DC) to migrate into the tumor, while reducing the number of immunosuppressive cells such as regulatory T cells (Treg) and myeloid-derived suppressor cells (MDSC) within the tumor microenvironment (TME). Single agent anti-SEMA4D shifted the balance of immune activity in the TME and significantly delayed tumor growth, but induced a relatively low frequency of complete tumor regression in some preclinical models. In contrast, when administered in combination with immune checkpoint inhibitors, anti-SEMA4D significantly enhanced the activity of anti-CTLA-4 and anti-PD-1 therapies [1]. The humanized IgG4 anti-SEMA4D was well-tolerated in phase I trials (NCT01313065) [2] and phase Ib/II studies of anti-SEMA4D in combination with anti-PD-L1 for the treatment of non-small cell lung cancer (NSCLC) are planned in collaboration with EMD Serono. Additional phase Ib/II studies of anti-SEMA4D in combination with anti-PD-L1 and/or anti-CTLA-4 for treatment of melanoma and head and neck squamous cell carcinoma (HNSCC) are anticipated.

Idera’s Oncology Lead, Mark Cornfeld, MD, MPH, presented an overview of the mechanism of action of IMO-2125, an investigational intratumoral toll-like receptor nine (TLR9) agonist that can modulate the TME to enhance anti-tumor immunity. IMO-2125 is specifically designed to activate TLR9, an immune signaling protein. Through TLR9, IMO-2125 activates DC and induces an innate immune response in the TME. The subsequent recruitment and activation of tumor infiltrating lymphocytes (TIL) and other immune cells enhances antigen presentation and T cell expansion. In previously completed clinical trials in the setting of hepatitis C infection, subcutaneous administration of IMO-2125 was generally well tolerated and had pharmacologic activity. Idera subsequently conducted extensive preclinical research in multiple animal models of cancer that showed intratumoral IMO-2125 enhanced the anti-tumor activity of checkpoint inhibitors. Data from these clinical and nonclinical studies supported the initiation of a phase I/II clinical trial of intratumoral IMO-2125 in combination with ipilimumab in patients with metastatic melanoma refractory to prior anti-PD-1 therapy. Preliminary data from this trial were first presented at the SITC 2016 Annual Meeting [3]. Results showed that escalating doses of IMO-2125 in combination with ipilimumab were well tolerated with a maximum tolerated dose not yet identified. DC maturation was observed in tumor biopsies obtained 24 h after the first IMO-2125 treatment and before ipilimumab treatment was initiated. In addition, immunologic activity in responding patients included increased T cell infiltration in untreated tumors.

A new approach, described by Edward Cha, MD, PhD (Genentech), sought to combine the efficacy of targeted therapies with the durable responses seen following immune checkpoint inhibition therapy. The rationale for combining the selective MEK1 and MEK2 inhibitor cobimetinib with the PD-L1 blocking drug atezolizumab arose from the observation that MEK inhibition has positive immunomodulatory effects including intratumoral T cell accumulation and upregulation of MHC class I, potentially promoting antigen presentation and tumor immunogenicity. Moreover, the combination of MEK inhibition and anti-PD-L1 led to enhanced efficacy and durable regression in multiple tumor models [4]. Tumor samples from the phase Ib study of the combination of cobimetinib and atezolizumab in patients with solid tumors reiterated the T cell and MHC class I effects of MEK inhibition seen in pre-clinical models, and preliminary data demonstrated a manageable safety profile in the population of metastatic colorectal cancer (CRC) patients. Of the CRC patients, four had confirmed partial responses (PR; per RECIST v1.1), three of which had known microsatellite-stable tumors, and responses for two of those patients were ongoing beyond 15 months. The durability of this treatment combination was also demonstrated in a cohort of patients with metastatic melanoma of cutaneous and mucosal origin. Among the 20 patients, there were nine (45%) confirmed PR with a median duration of response of 15 months [5].

In another discussion of novel combination strategies, the Chief Medical Officer for Celldex Therapeutics, Inc., Thomas Davis, MD, led with the assertion that it will be necessary to influence multiple steps of the immune response in order to maximize the clinical benefits of immunotherapy, a sentiment that was echoed by several presenters throughout the program. Towards that goal, a number of agents in the Celldex pipeline aim to impact the recruitment and activation of dendritic cell (DC) or T cells. Examples of these immunotherapeutics include CDX-1401, CDX-301, and varlilumab. CDX-1401 is an antibody fusion protein that delivers antigen to DC by targeting DEC-205, which can efficiently internalize and present any antigen attached to the antibody. Phase I studies of this antigen delivery system using NY-ESO-1 as the fusion antigen given in combination with poly-ICLC showed excellent tolerability and generated good immune responses. The establishment of a tumor-specific immune response significantly increased patient responsiveness to subsequent immune checkpoint blockade in 7/7 (100%) patients with NY-ESO-1+ tumors. The CDX-1401 vaccine is being tested in combination with CDX-301 (Flt3L), a potent expander of DC progenitors [6], via collaboration with the Cancer Immunotherapy Trials Network (CITN). Varlilumab is another Celldex product candidate that was mentioned briefly. This CD27 agonist is a potent lymphocyte activator with essentially no associated toxicity and is currently being investigated in different combinations, including with CDX-1401 and immune checkpoint inhibitors.

Rom S. Leidner, MD (Earle A. Chiles Research Institute) discussed the vital role that natural killer (NK) cells play in immune surveillance and control of tumor growth. NK cell activation is regulated in part by killer cell immunoglobulin-like receptors (KIR), predominantly expressed on NK cells as well as some CD8+ T cells. Lirilumab is a fully human IgG4 monoclonal antibody that targets inhibitory KIR, thereby promoting NK cell antitumor activity. Potentiating an antitumor immune response through blocking inhibitory KIR may complement other immuno-oncology therapies that enhance T cell activity, such as the immune checkpoint inhibitors nivolumab (anti-PD-1) and ipilimumab. Dr. Leidner presented safety data from two phase I studies of lirilumab in combination with nivolumab (CA223-001; NCT01714739) or ipilimumab (CA223-002; NCT01750580) in patients with advanced solid tumors. In the dose-escalation and cohort-expansion phases of the ongoing CA223-001 study, 159 patients were treated with lirilumab 0.1 to 3 mg/kg every 4 weeks (Q4W) plus nivolumab 3 mg/kg Q2W. In the dose-escalation phase of CA223-002, 22 patients were treated with lirilumab 0.1 to 3 mg/kg Q3W plus ipilimumab 3 mg/kg Q3W. Both combination regimens were manageable. The safety profile of lirilumab plus nivolumab or ipilimumab appeared consistent with prior reports of nivolumab or ipilimumab monotherapy, with the exception of low-grade infusion-related reactions with lirilumab plus nivolumab; these events were manageable, and most occurred after the first dose. Further evaluation of lirilumab plus nivolumab is ongoing.

Another approach targeting NK cells was presented by Adi Diab, MD (The University of Texas MD Anderson Cancer Center). NKTR-214 is a CD122-biased agonist and is the only cytokine immunotherapy that preferentially expands both effector CD8+ T cells and NK cells within the TME. Preclinical data show tumor growth suppression in multiple tumor models when used as a single agent or in combination. A phase I/II trial was initiated to evaluate the safety and efficacy of NKTR-214 and to assess immune changes in the TME. Patients with locally advanced or metastatic solid tumors were administered NKTR-214 IV q2-q3 weeks as a 15 min IV infusion starting at a dose of 0.003 mg/kg. As of November 9, 2016, 25 patients had received treatment with NKTR-214 in 5 different dose cohorts ranging from 0.003 mg/kg-0.012 mg/kg. The median number of prior anticancer therapies was two and 60% of patients had received at least one prior immunotherapy agent. One patient experienced dose-limiting toxicities (DLT) (Grade 3 syncope and hypotension) at 0.012 mg/kg. No immune-related adverse events (AE) or capillary leak syndrome were observed at any dose. There have been no grade 4 toxicities or deaths on study; 4/25 (16%) experienced a Grade 3 treatment emergent AE and 3/25 (12%) experienced Grade 3 hypotension. All cases of hypotension were rapidly reversed with fluids and no patient discontinued treatment as a result. In all patients evaluated, analysis of blood samples showed concordant increases in Ki67+ immune cells, PD-1+ CD8+ T cells, and NK cells 8 days after a single dose of NKTR-214. Flow cytometry enumeration and/or immunohistochemistry (IHC) revealed an up to 10-fold increase from baseline in CD8+ T cells and NK cells in the TME, with minimal changes to Treg. Demonstrating encouraging evidence of single agent activity in heavily pre-treated patients, 7/18 (39%) patients had radiographic tumor reductions and one patient with renal cell carcinoma (RCC) had an unconfirmed partial response (PR) at the initial 6 or 8-week scan.

Closing the Clinical session, Patrick Soon-Shiong, MD (NantWorks/NantKwest) discussed a novel line of off-the-shelf activated NK cells called NK-92. This platform makes use of NK cells as key rapid-responders to more efficiently target malignant cells and has moved from phase I to phase II trials. Specifically, the NK-92 cell line is armed with activation receptors, but lacks inhibitory receptors and can be further engineered to target tumor cells in a customizable way. When made to express the high affinity Fc receptor CD16, NK-92 can help mediate antibody killing in combination with so-called “chimeric antigen receptors (CAR) in a bottle” such as anti-HER2, anti-CD20, and anti-EGFR. Alternatively, NK-92 can be made to bind directly to target antigens via expression of CAR. Data from studies of the high affinity CD16-expressing NK-92 in combination with the IgG1-based agents trastuzumab, pertuzumab, cetuximab, and avelumab for the treatment of breast cancer and lung cancer, have demonstrated the impressive tumoricidal activity of the high affinity NK-92 technology. Additionally, a targeted version of single agent NK-92 was given to a heavily pretreated patient with advanced Merkel cell carcinoma and a response was noted within 14 days of NK-92 infusion, with radiological CR by day 171. Using this platform, pre-clinical investigations of NK-92/5.28.z cells targeting ErbB2 have been shown to induce secondary responses in a murine model of glioma [7], which provides an encouraging framework from which to move into clinical trials in the setting of highly metastatic disease.

## Pre-Clinical New Agents in Development

Bryan A. Irving, PhD (CytomX Therapeutics, Inc.) kicked off the Pre-Clinical session with a presentation on T cell-engaging bispecific antibodies (TCB), which represent a highly potent vehicle for directing the activity of cytotoxic T cells against tumors, including tumors that lack sufficient mutations to generate tumor-specific immunity. TCB have shown clinical activity in hematologic malignancies but their development for non-hematologic cancers has been challenging, due in part to toxicities that result from interaction with healthy cells expressing the target antigen. Therefore, new approaches are needed that enable the use of TCB without on-target damage to normal tissues. CytomX has developed a new class of antibodies, proteolytically activatable antibody prodrugs called Probody™ therapeutics that are designed to widen the therapeutic window by minimizing interaction with normal tissue and maximizing interaction with tumor tissue. Probody therapeutics are “masked” to reduce binding to antigen in healthy tissue, but can become “unmasked” in the TME by tumor-specific protease activity. CytomX has demonstrated the ability of a Probody T cell-engaging bispecific (Pb-TCB) targeting CD3 and epidermal growth factor receptor (EGFR), to provide equivalent anti-tumor activity in NSG (NOD scid gamma) mice to its corresponding unmasked antibody bispecific, while increasing the maximum tolerated dose and exposure by >30-fold and 300-fold, respectively, in cynomolgus monkeys. By localizing their activity to the TME, Pb-TCB have the potential to expand clinical opportunities for T cell-engaging bispecific therapies in solid tumors, which are currently limited by on-target toxicities.

Shane A. Olwill, PhD (Pieris Pharmaceuticals GmbH), presented data on CD137 (4-1BB), a key costimulatory immunoreceptor and a highly promising therapeutic target in cancer. To overcome limitations of current monoclonal antibody (mAb)-based approaches which target CD137 monospecifically, a CD137/HER2 bispecific (PRS-343) was designed to promote CD137 clustering by bridging CD137-positive T cells with HER2-positive tumor cells, thereby providing a potent costimulatory signal to tumor antigen-specific T cells. PRS-343 was generated as a genetic fusion of a CD137-specific Anticalin® protein to an IgG4 variant of trastuzumab. PRS-343 was found to efficiently activate T cells ex vivo in the presence of HER2-positive cells. In vivo proof of concept studies showed that PRS-343 led to strong tumor growth inhibition in a dose-dependent manner, compared to treatment with isotype control. Tumor response was accompanied by a significantly higher frequency of hCD45+ TIL as determined by IHC. T cell phenotyping indicated that the increase in TIL frequency was due to expansion of CD3+ CD8+ T cells, while CD4+ lymphocytes remained at a low frequency. PRS-343 was shown to elicit potent costimulatory T cell engagement of the immunoreceptor CD137 in a HER2-dependent manner, and to display dual activity in vivo, based on monospecific HER2-targeting and bispecific, tumor-localized costimulation of CD137. Compared to known CD137-targeting antibodies in clinical development, PRS-343 may provide more localized activation of the immune system with higher efficacy and reduced peripheral toxicity. The positive functional data of PRS-343 support investigation of its anti-cancer activity in clinical trials.

Thomas W. Dubensky Jr., PhD (Aduro Biotech) discussed a personalized therapy in development, known as pLADD, which is live, attenuated double-deleted *Listeria monocytogenes* (LADD) that has been engineered to encode multiple tumor-specific neoantigens. The LADD platform is an attractive approach for personalized immunotherapy due to the rapid construction, manufacture, and release of pLADD clinical strains. Moreover, a clinical safety and efficacy profile has been established in over 400 patients and the robust activation of innate immunity and TME remodeling has been demonstrated in preclinical models and in patients. The Aduro Biotech group partnered with Hanlee P. Ji, MD, PhD (Stanford University), a physician-scientist focused on colorectal cancer (CRC) and has developed proprietary computational methods for neo-epitope identification [8]. In preclinical models, novel methods were developed to site-specifically integrate expression cassettes into the pLADD chromosome, resulting in the robust expression, secretion and processing of approximately 25 encoded neoepitopes into the MHC class I presentation pathway of infected antigen presenting cells. Subsequent studies using tumor-bearing mice demonstrated that a pLADD strain expressing tumor-specific neoepitopes from murine MC38 tumor cells could induce robust CD8+ T cell responses specific for encoded neoepitopes, but not against native sequences. This personalized approach was highly effective in combination with PD-1 blockade. An Investigational New Drug Application has been allowed, and a phase I trial will be initiated in 2017 to evaluate the safety and immunogenicity of pLADD in patients with cancers of the gastrointestinal tract, focusing on microsatellite stable (MSS) CRC, an indication in which responses with immune checkpoint inhibition monotherapy have been poor.

The presentation by David Tuck, MD (Curis) focused on CA-170. This small molecule is an orally bioavailable antagonist of the PD-L1, PD-L2, and VISTA/PD-1H immune checkpoint pathways and is currently undergoing phase I clinical testing. CA-170 was developed through a rational design and screening strategy that identified small molecules capable of antagonizing T cell suppression mediated by PD-L1, PD-L2, and VISTA/PD-1H in vitro. CA-170 exhibits potent immune rescue activity, comparable to that of PD-1 or VISTA/PD-1H blocking antibodies in functional assays. CA-170 does not exhibit off-target activity against CTLA-4, LAG-3, BTLA pathways, or the B7/CD28 pathway. In immune-competent mice, orally administered CA-170 inhibits the growth of syngeneic tumors, enhances peripheral T cell activation, and promotes the activation of tumor infiltrating CD8+ T cells in a dose-dependent manner. Pre-clinical safety studies of CA-170 in rodents and non-human primates showed no signs of toxicity when dosed orally up to 1000 mg/kg for 28 consecutive days. CA-170 exhibits an oral bioavailability of approximately 40% and <10% in mouse and cynomolgus monkey, respectively, with respective plasma half-lives ranging from 0.5 h to 3.25-4.0 h. The clinical pharmacokinetic profile is similar to non-clinical and human exposure and appears to be highly predictable on oral dosing. CA-170 leads to increases in activated CD8+ T cells in the peripheral blood of cancer patients following oral dosing, supporting its continued clinical development.

Frederic Triebel, MD, PhD (Prima Biomed) shared pre-clinical data for IMP321, which is a LAG-3Ig fusion protein that binds to major histocompatibility complex (MHC) class II molecules on the surface of APC and triggers activation of APC and CD8+ T cells known to mediate tumor recognition and killing. IMP321 induces more Tc1 subset differentiation and IFN-γ compared to other APC activators, such as CD40L, or TLR agonists that induce immunosuppressive IL-10 production [9]. In the clinic, IMP321 has been used at low doses as an adjuvant to cancer vaccines [10–12] and at higher doses as an APC activator to boost the DC network loaded with tumor antigens after first-line chemotherapy [13]. The randomized, double blind, placebo-controlled AIPAC (ACTive Immunotherapy PAClitaxel) phase IIb registration trial has now commenced in the EU (enrolling 241 patients) and will test IMP321 combined with weekly paclitaxel in a first-line setting in hormone receptor-positive metastatic breast cancer (NCT02614833). Inducing more TIL at the tumor site with an APC activator like IMP321, while releasing the PD-1 brake on TIL, may lead to greater anti-tumor efficacy than anti-PD-1 agents alone. Synergistic activity of the LAG-3Ig/anti-PD-1 combination has been shown pre-clinically in human peripheral blood mononuclear cells (PBMC) from 10 donors stimulated with cytomegalovirus peptides, and in a CT26wt colon cancer mouse model. The phase I TACTI-mel (Two ACTive Immunotherapies in Melanoma) trial, initiated in 2016, is examining the combination IMP321 + pembrolizumab in unresectable or metastatic melanoma (NCT02676869).

To treat solid tumors, the laboratory of Prasad S. Adusumilli, MD, FACS, FCCP (Memorial Sloan Kettering Cancer Center) has chosen mesothelin, a cell-surface antigen, as a target for CAR T cell therapy. Mesothelin is expressed in the majority of solid tumors and is associated with aggressive cancer growth, thus serving as a rational target [14]. The group demonstrated that regional delivery of mesothelin-targeted, second-generation CAR T cells achieved CD4+ T cell-dependent, long-term immunity even at a 30-fold lower dose than systemically delivered CAR T cells [15]. They translated these observations to two clinical trials. In the first trial (NCT02414269), mesothelin-targeted CAR T cells were administered intrapleurally to patients with mesothelioma, lung, or breast cancer with pleural disease, and in the second trial (NCT02792114), CAR T cells were administered systemically to patients with HER-2-negative metastatic breast cancer. To overcome tumor-mediated inhibition of CAR T cells, they developed and evaluated T cell extrinsic (PD-1 blocking antibody) and intrinsic (co-transduction of CAR T cells with a PD-1 dominant negative receptor (DNR) or PD-1/4-1BB fusion protein) strategies to overcome PD-L1/2 inhibition. The addition of PD-1 blocking agents does potentiate CAR T cell therapy but multiple administrations are necessary. By contrast, a single dose of mesothelin-targeted CAR T cells co-expressing PD-1 DNR restores effector functions, enhances tumor control, and prolongs median survival [16]. Converting PD-L1 inhibition into a positive costimulatory signal by PD-1/4-1BB construct co-transduction into CAR T cells enhanced cytokine secretion and T cell accumulation. These strategies to prolong the functional persistence of CAR T cells are now being investigated in clinical trials.

Wrapping up the Pre-Clinical session, Jane Grogan, PhD (Genentech) presented on the immunoreceptor TIGIT (T cell immunoreceptor with Ig and ITIM domains), which was originally discovered nearly a decade ago. An inhibitory receptor found on T cells and NK cells, TIGIT acts synergistically with the PD-1/PD-L1 axis upon binding to its cognate receptor PVR, which is expressed on tumor cells or DC [17]. The result of this interaction serves to limit T cell activity within the TME. Pre-clinical models of established tumors treated using combined blockade of both the TIGIT/PVR and PD-1/PD-L1 axes demonstrate superior rescue of exhausted or anergic T cells compared with inhibition of either pathway alone [18]. TIGIT is thought to regulate anti-tumor T cell effector responses in a number of different ways. Due to the presence of its ITIM-like domain, there is capacity for TIGIT to signal in *cis* into a cell and shut down T cell responses, although this has not yet been shown in primary cells [17, 19–24]. In vivo and in vitro models have also shown that engagement of PVR by TIGIT is sufficient to downregulate the production of inflammatory IL-12 by DC and upregulate TGFβ and IL-10 production, which could reinforce an immunosuppressive TME [17]. Additionally, the relatively higher affinity of TIGIT for PVR competes with the lower affinity activating PVR ligand, CD226, and can replace it in the synapse, thereby supporting T cell inhibition. Together with the discovery of high TIGIT expression in the TIL and peripheral blood of patients with NSCLC, these data support moving an immunotherapy agent targeting TIGIT into phase I clinical trials.

## Strategic Considerations of Combinations and Biomarkers in New Agent Development

### Combination Immunotherapy Approaches

Although the greater than 800 open trials of immunotherapy combinations currently underway [25] might seem overwhelming, Charles G. Drake, MD, PhD (Columbia University Herbert Irving Comprehensive Cancer Center) suggested that this number seemed low when taking into account all of the combinations possible. Laying the foundation for the Extended Panel Discussion which addressed how to prioritize among so many therapeutic candidates, Dr. Drake highlighted combinations that are already FDA-approved, before going on to discuss the possibility of combining immunotherapy with conventional approaches, targeting other cell populations such as myeloid cells, and using immune activators, by way of illustrating the plethora of possibilities for combination strategies based on cancer immunotherapy.

To date, combinatorial approaches that have received FDA approval are limited to combinations of single immunotherapeutic agents, such as ipilimumab + nivolumab in the setting of unresectable or metastatic melanoma [26]. The biological rationale for this strategy is that the molecules targeted may act on different cell types within the TME. That is, PD-1 operates at the junction between the T cell and the tumor cell or APC, deactivating the T cell. In contrast, CTLA-4 is predominantly expressed by Treg in the TME, where it exerts contact-dependent suppression. Despite the synergism predicted by animal models, ipilimumab + nivolumab has only proven additive in humans. Dr. Drake cautioned that this additive efficacy comes with ipilimumab-driven additive toxicity [27]. Moreover, this approach has led investigators to revisit the topic of predictive biomarkers, since PD-L1 positivity did not correlate with clinical outcomes. Other attempts to take advantage of the synergism of combination immunotherapy observed in animal models have targeted multiple immune checkpoints on the same cell, such as PD-1 and LAG-3. T cells that co-express more than one immune checkpoint tend to be the least functional, and co-blockade of these molecules in animal models has resulted in synergistic effects [28] across multiple combinations of agents, but this has not yet been shown in humans.

The ways in which conventional chemotherapy affect the immune system will influence strategies for combining these drugs with immunotherapy agents. For example, chemotherapy can result in immunogenic cell death, accompanied by release of tumor antigens, destruction of immunosuppressive populations including MDSC and M2 macrophages, and acquisition of effector function due to lymphopenia-induced homeostatic proliferation [29]. One implication of this is the need to consider timing of drug administration, as PD-1 blockade may be most important at the time of antigen encounter (chemotherapy-induced immunogenic cell death) [30]. When considering combining immunotherapy with standard cancer treatments, it is tempting to think first about chemotherapy and radiation. However, Dr. Drake shared preliminary evidence that more attention should be paid to other anti-tumor agents, such as hormonal therapy. Trials of anti-PD-1/PD-L1 monotherapy in patients with prostate and colorectal cancer did not lead to encouraging objective responses [31], perhaps owing to a lack of anti-PD-1/PD-L1 therapy targets in the TME. Interestingly, investigations of patients with castration resistant prostate cancer that progressed on enzalutamide revealed that enzalutamide resistance is associated with the expression of PD-1 and PD-L1/2 on antigen presenting cells [32], and the addition of anti-PD-1 in a small trial of such patients resulted in durable objective responses as well as reductions in PSA levels [33]. Another therapeutic that has yielded exciting results in combination with immunotherapy is VEGF inhibition. In one recent study of atezolizumab (anti-PD-L1) + bevacizumab (anti-VEGF) in kidney cancer, overall response rate was 40% (historical response rates with atezolizumab and bevacizumab monotherapy are approximately 15 and 9%, respectively) and a tolerable safety profile [34]. It is postulated that VEGF blockade may function in coordination with anti-PD-L1 by normalizing the tumor vasculature, which would facilitate trafficking of T cells into the TME [34], although it has also been shown to promote immunogenic cell death, and generation of suppressive DC and MDSC [35].

Other rational combination approaches seek to address the hostile TME. A number of these trials involve inhibition of indoleamine 2,3-dioxygenase (IDO), an enzyme produced by MDSC and dysfunctional DC within the TME that leads to generation of Treg, which further reinforce an immunosuppressive environment [36]. The population of IL-10-secreting suppressive macrophages found in tumors appear to be maintained by CSF-1, which has also become an attractive therapeutic target [37], primarily in combination with immune checkpoint blockade. In the case of tumors that are “immunological deserts” [38] such as prostate cancer, which is typically poorly infiltrated by T cells, one other approach is to introduce an agent intratumorally (e.g. talimogene laherparepvec, viral constructs, TLR agonists, etc.) that can activate the TME to make it more visible to the immune system. Together with immune checkpoint inhibition to overcome adaptive immune resistance, this approach has the potential to lead to an abscopal effect by increasing direct presentation of tumor antigens to T cells at the site of the primary tumor as well as cross-presentation in the draining lymph node. This could generate a population of activated tumor-specific CD8+ T cells that traffic throughout the body and provide systemic control. Although the majority of these combination approaches show promise in animal models, choosing an effective combination to move into human clinical trials remains a challenge due to the inherent disparities between animal models and humans.

### Extended Panel Discussion

The multidisciplinary panel, moderated by Dr. Chen, comprised Dr. Bot, Dr. Dansey, Dr. Drake, Gordon J. Freeman, PhD (Dana-Farber Cancer Institute), Raj K. Puri, MD, PhD (U.S. FDA), and Marc Theoret, MD (U.S. FDA). Dr. Chen opened the discussion by asking panel members to describe their vision of the future, considering the large number of combination immunotherapy approaches currently in clinical trials. The heterogeneity of malignant diseases along with cost and safety concerns will continue to drive the need for multiple solutions. Contributing to the complexity of this issue is the fact that there are several different ways to give two drugs together, a challenge that increases significantly with the introduction of a third agent. Panel members uniformly expressed optimism about the influx of potential new treatment strategies and saw this as an opportunity to determine which strategies best achieve the balance of efficacy and safety, and to establish the mechanistic basis for efficacy.

To rapidly prioritize among the combination trials and ensure that only the most promising trials are selected to move forward, it will be critical to integrate biomarkers into clinical decision-making. For example, although biomarkers predictive of response to treatment remain incompletely defined for immunotherapeutics, there are populations known to be highly responsive to such treatments, including people with microsatellite instability, those with *PDL1* amplifications, and the presence of human papillomavirus, Epstein-Barr virus, or the Merkel cell polyomavirus in patients whose tumors have a strong viral etiology. Additionally, optimization of the drug development process and collaboration across disciplines were identified as opportunities to improve trial design on the front end of the process.

The recent influx of combination trials would seem to represent a burden for the FDA, but Dr. Theoret explained that the current regulatory framework is designed with two approval pathways to help facilitate the process as efficiently as possible. Agents that demonstrate substantial evidence of a treatment effect that is representative of clinical benefit in adequate and well-controlled clinical trials are evaluated via the regular approval pathway, which does not require demonstration of comparative efficacy. In contrast, all four of the FDA expedited programs, which includes an accelerated approval pathway, do consider the available therapy. Intended for therapies that address unmet medical needs for serious and life-threatening conditions, the expedited programs are set up to facilitate and expedite the development of agents at multiple points in the process.

## Conclusions

The concepts and strategies presented at the SITC 2016 New Cancer Immunotherapy Agents in Development program highlighted creative and elegant approaches in a rapidly-evolving field. A common thread throughout all presentations was a need for a deeper understanding of the mechanisms by which current immunotherapies exert their effects in order to continue to improve cancer outcomes. Mechanistic approaches can then potentially lead to rational combinations of two or more agents that exert an immunologic effect as well as provide potential predictive biomarker candidates to help identify those patients most likely to benefit from a particular approach. Many presenters also expressed the opinion that no single agent was likely to be the “magic bullet” that has been long sought-after in the fight against cancer; rather, combination approaches that provide a multi-pronged intervention are expected to yield the greatest clinical success.

## Abbreviations

AE: Adverse event(s)
APC: Antigen presenting cell(s)
CAR: Chimeric antigen receptor(s)
CITN: Cancer Immunotherapy Trials Network
CRC: Colorectal cancer
DC: Dendritic cell(s)
DLT: Dose-limiting toxicity
DNR: Dominant negative receptor
EGFR: Epidermal growth factor receptor
FDA: Food and Drug Administration
HNSCC: Head and neck squamous cell carcinoma
IDO: Indoleamine 2,3-dioxygenase
IHC: Immunohistochemistry
KIR: Killer cell immunoglobulin-like receptor(s)
LADD: Live-attenuated double-deleted *Listeria monocytogenes*
mAb: Monoclonal antibody
MDSC: Myeloid-derived suppressor cell(s)
MHC: Major histocompatibility complex
MSS: Microsatellite stable
NK: Natural killer
NSCLC: Non-small cell lung cancer
PBMC: Peripheral blood mononuclear cells
PFS: Progression-free survival
PR: Partial response
RCC: Renal cell carcinoma
SEMA4D: Semaphorin 4D
SITC: Society for Immunotherapy of Cancer
TCB: T cell-engaging bispecific antibodies
TEAE: Treatment-emergent adverse event(s)
TIL: Tumor infiltrating lymphocyte(s)
TLR: Toll-like receptor
TME: Tumor microenvironment
Treg: Regulatory T cell(s)
